# Supplementary material for: Sorting at embryonic boundaries requires high heterotypic interfacial tension
Source: Nat Commun. 2017 Jul 31;8:157. doi: 10.1038/s41467-017-00146-x (PMC5537356; doi:10.1038/s41467-017-00146-x)
Supplement: Supplementary file 2 — Supplementary Software 1 [file 41467_2017_146_MOESM2_ESM.zip › PottsModel/SrcPottsModel/doc/index-files/index-13.html]

M-Index


JavaScript is disabled on your browser.


Skip navigation links


- Overview
- Package
- Class
- Use
- Tree
- Deprecated
- Index
- Help

- Prev Letter
- Next Letter

- Frames
- No Frames

- All Classes

A B C D E F G H I J K L M N O P R S T U V W 


## M

main(String[]) - Static method in class engine.CommandLineSimulation


main(String[]) - Static method in class gui.PottsCanvas


main(String[]) - Static method in class gui.Utils


main(String[]) - Static method in class model.LatticeUtilities


main(String[]) - Static method in class simulations.BoundarySimulation


main(String[]) - Static method in class simulations.DefaultSimulation


main(String[]) - Static method in class simulations.DITHSimulation


main(String[]) - Static method in class simulations.EndoSimulation


main(String[]) - Static method in class simulations.FourByFourSimulation


main(String[]) - Static method in class simulations.GenerateRandomLattices


main(String[]) - Static method in class simulations.GranerGlazierSimulation


main(String[]) - Static method in class simulations.NegativeControl


main(String[]) - Static method in class simulations.SingleCell


main(String[]) - Static method in class utils.HSLColor


main(String[]) - Static method in class utils.IO


main(String[]) - Static method in class utils.JavaVersion


makeRound(int[][][], int) - Static method in class model.LatticeUtilities


matrixToString(int[][]) - Static method in class utils.IO


maxTemperature - Variable in class model.Constants.ParameterManager


mean(List<Double>) - Static method in class engine.Statistic.Utils


mean(float[]) - Static method in class engine.Statistic.Utils


mean(EnergyFunction.Energy) - Method in class engine.Utils.EnergyTracker


measureAngle(Point) - Method in class model.FiniteVector


measureAngle(Point) - Method in class model.HalfLine


measureAngle(Point) - Method in interface model.Vector


measureAngle(Vector, Point) - Static method in class model.VectorUtils


median(LinkedList<Double>) - Static method in class engine.Statistic.Utils


minTemperature - Variable in class model.Constants.ParameterManager


model - package model


model - Variable in class model.Constants.ParameterManager


mvc - package mvc

A B C D E F G H I J K L M N O P R S T U V W

Skip navigation links


- Overview
- Package
- Class
- Use
- Tree
- Deprecated
- Index
- Help

- Prev Letter
- Next Letter

- Frames
- No Frames

- All Classes
